# A Rising Cancer Prevention Target of RSK2 in Human Skin Cancer

**DOI:** 10.3389/fonc.2013.00201

**Published:** 2013-08-05

**Authors:** Narayanasamy Arul, Yong-Yeon Cho

**Affiliations:** ^1^College of Pharmacy, The Catholic University of Korea, Bucheon-si, Republic of Korea

**Keywords:** RSK2, skin cancer, carcinogenesis, chemoprevention, RSK2 inhibitors

## Abstract

RSK2 is a p90 ribosomal S6 kinase family (p90^RSK^) member regulating cell proliferation and transformation induced by tumor promoters such as epithelial growth factor (EGF) and 12-*O*-tetradecanoylphorbol-13-acetate. This family of p90^RSK^ has classified as a serine/threonine kinase that respond to many growth factors, peptide hormones, neurotransmitters, and environmental stresses such as ultraviolet (UV) light. Our recent study demonstrates that RSK2 plays a key role in human skin cancer development. Activation of RSK2 by EGF and UV through extracellular-activated protein kinases signaling pathway induces cell cycle progression, cell proliferation, and anchorage-independent cell transformation. Moreover, knockdown of RSK2 by si-RNA or sh-RNA abrogates cell proliferation and cell transformation of non-malignant human skin keratinocyte, and colony growth of malignant melanoma (MM) cells in soft agar. Importantly, activated and total RSK2 protein levels are highly detected in human skin cancer tissues including squamous cell carcinoma, basal-cell carcinoma, and MM. Kaempferol and eriodictyol are natural substances to inhibit kinase activity of the RSK2 N-terminal kinase domain, which is a critical kinase domain to transduce their activation signals to the substrates by phosphorylation. In this review, we discuss the role of RSK2 in skin cancer, particularly in activation of signaling pathways and potent natural substances to target RSK2 as chemopreventive and therapeutic agents.

## Introduction

Skin cancer is the most common of all cancer types in human. Over two-million new cases of skin cancer are diagnosed annually in the United States (U.S.) and this accounts for over 40% of all new cancer cases (1). Skin cancer rate in the U.S. is increasing because of the ultraviolet (UV)-light influence by the depletion of ozone layer of atmosphere and aging population.

Skin cancer is categorized by outermost layer of the skin called epidermis; it is composed of three layers: an upper and the middle layers are made up of squamous cells, and the bottom layer is made up of melanocytes and basal cells. Basal-cell carcinoma (BCC) is the most common form of skin cancer accounting for more than one million cases which were diagnosed in U.S. during the year 2009 (2). Squamous cell carcinoma (SCC) accounts for 16–20% of skin cancer cases and occurs twice as often in men than in women. Although basal-cell carcinoma rarely metastasizes, it can cause significant destruction and disfigurement by invading surrounding tissues, so it is considered to be malignant (3, 4). According to U.S. statistics report, approximately 3 out of 10 Caucasians may develop BCC in their life period (5). The most common skin cancer is BCC affecting one in seven individuals over a life period in Canada (6). In 80% of all cases, BCC is found on the head and neck (5). Melanoma (from Greek melas mean “dark”) is a malignant tumor of melanocytes in any part of the body (3, 4). Melanocytes occur not only in skin, but also in other parts of the body, including the bowel and the eye, and produce the dark pigment, melanin, which is responsible for the color of skin. Although melanoma is less common than other skin cancers, it is more dangerous if it is not diagnosed early because it causes about 75% of the majority of deaths related to skin cancer (5, 7). In worldwide, approximately 160,000 new cases of melanoma are diagnosed annually.

The main risk factor for non-melanoma skin cancer is sunlight (1). The UV part of sunlight is subdivided according to wavelength-UVA (320–400 nm), UVB (280–320 nm), and UVC (200–280 nm). Generally UV light on the earth surface contains 90–99% of UVA and 1–10% of UVB according to geographical location, season, and weather condition (8). UVC is usually absorbed in ozone layer of the earth atmosphere. UVB irradiation induces DNA damage, which can be replicated to generate mutations in many genes containing tumor suppressor *TP53* and *Ras* (1). This process generally designated as “initiation”; so UVB can act as an initiator. UVA generates heat and induces activation of diverse cellular signaling pathways. Because of UVA’s weak initiating activity, UVA is known to as a potent “promoter.” Based on these reasons, sunlight is a most abundant complete carcinogen for the skin cancer initiation and promotion in our environment.

The mitogen activated protein kinase (MAPK) signaling pathways plays a central role in diverse cancer development in human. Growth factors, cytokines, and environmental stresses such as UV activate receptor tyrosine kinases in cytoplasmic membrane (1, 8). The activated signals in the cytoplasmic membrane are transmitted to nucleus through phospho-conveyer systems including MAPK signaling pathways, which are composed of extracellular-activated protein kinases (ERKs), p38 kinases, and Jun N-terminal kinases (JNKs) (8). The signaling induces *c-fos* gene expression and phosphorylation of c-Jun at Ser63 and Ser73 (Ser63/73), resulting to form a Jun/Fos dimer (AP-1 transcription factor complex) (1, 8). About over 50% of cellular genes are regulated their gene expression by AP-1, particularly genes involved in cell proliferation, transformation, and cancer development (1).

The p90^RSK^ (ribosomal protein S6 kinase: RSK) is a family of 90 kDa serine/threonine kinases, which are composed of N-terminal domain (NTD), linker region (LR), C-terminal domain (CTD), and two kinase domains designated as N-terminal kinase domain (NTKD) and C-terminal kinase domain (CTKD) (9–11). ERKs, which can be activated by stimulation of growth factors, cytokines, and/or environmental stresses through a phosphorylation cascade system, activate RSKs including RSK1, RSK2, RSK3, MSK1, and MSK2. RSKs play an important role in activation of downstream transcription factors involved in cell proliferation, transformation, and cancer development (12–19). Importantly, RSK2 is genetically and physiologically linked with human genetic disease known as Coffin–Lowry Syndrome (CLS), but not in RSK1, RSK3, MSK1, and MSK2, indicating that RSK2’s physiological function is not redundant with other RSKs isotypes (20). Moreover, extensive studies on the RSK2 function in cell proliferation, transformation, and cancer development have demonstrated that RSK2 is an important kinase involved in human skin cancer development (16, 17, 19). In this review, we will discuss the role of RSK2 and a molecular target as a chemopreventive or therapeutic agent in human skin cancer.

## RSK2 Structure

**Figure 1:**
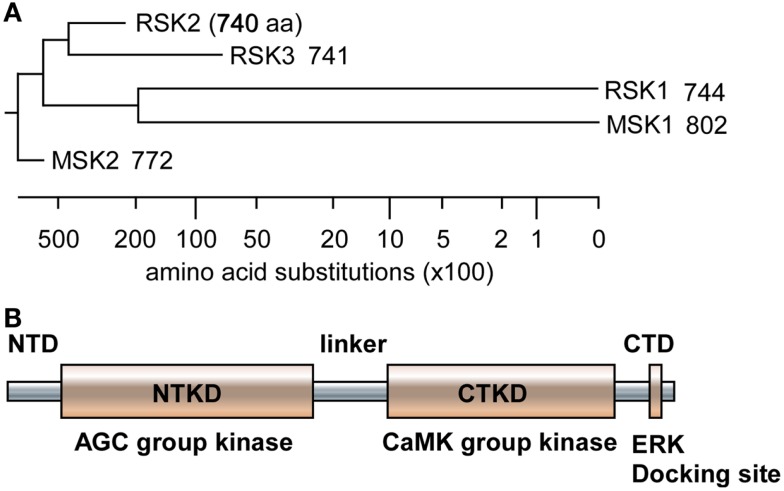
**Phylogenic tree and RSK2 domain structure**. **(A)** The amino acid sequences of human RSK1, RSK2, RSK3, MSK1, and MSK2 were downloaded from human protein reference data base, similarity was obtained by Jotun Hein method, and phylogenic tree was drawn using the DNASTAR computer program. **(B)** A domain structure of RSKs. RSKs, including RSK1, RSK2, RS3, MSK1, and MSK2, contain two kinase domains designated as N- and C-terminal kinase domain (NTKD and CTKD), N- and C-terminal domains (NTD and CTD), and a linker domain in a polypeptide. The NTKD belong to AGC kinase group kinases and the CTKD has been classified as CaMK group kinases. The CTD consists of an ERK docking site and the linker domain contains ERK-mediated and RSK2 auto-phosphorylation sites.

Since RSKs is discovered in *Xenopus laevis* oocytes by Erikson and Maller as a kinase to phosphorylate the 40S ribosomal subunit protein S6 (21–23), RSKs were classified into two subfamilies including RSKs, RSK1, RSK2, and RSK3, and MSKs, MSK1 and MSK2, based on the amino acid homology and functional identities (9, 22). The RSK subfamilies share about 80% amino acid homology, and MSKs subfamily shows about 60% of amino acid similarity in primary structure. In contrast, RSKs and MSKs share about 40% of amino acid similarity in primary structure (Table 1), suggesting that RSKs and MSKs might be functionally and physiologically separated. In addition, amino acid identities of RSKs between human and mouse indicates that human RSK1, RSK2, RSK3, MSK1, and MSK2 shows about 95% of amino acids similarity with the ortholog of each RSKs in mouse (Table 2), indicating RSK family members are functionally well conserved proteins between human and mouse. Hence phylogenic studies suggested by Hein and his colleagues (24) indicate that a group of the RSK1 and MSK1 is evolutionally distinct kinase group from the other kinase group including RSK2 and RSK3. Furthermore, MSK2 is branched from RSK1, RSK2, RSK3, and MSK1 is the earliest period in the evolution process (Figure 1A). One of the key characteristics of RSK family in structure is that RSKs contain two distinct kinase domains in a single polypeptide chain which has not been identified in cellular serine/threonine kinases of MAP kinases (Figure 1B). The NTKD belongs to an AGC group (PKA, PKG, and PKC) of kinase family, and CTKD is classified as a group of calcium/calmodulin-dependent (CaMK) kinase family. Recently, our research group has resolved key structural features of RSK2, NTKD, and CTKD by X-ray crystallography (25, 26). The structural analysis demonstrates that auto-inhibitory αL-helix of the RSK2 in CTKD embeds in the kinase scaffold and forms inactive kinase conformation (25). *In vitro* study of the RSK2 signaling pathway demonstrates that ERK1 and 2, but not p38 kinases, are direct upstream kinases to phosphorylate in the LR of RSK2 (17). The result strongly suggests that when RSK2 is activated by upstream signaling molecules such as ERKs, RSK2 leads to displacement of the αL-helix, resulting in the rearrangement and reorganization of the T-loop into the active confirmation (25). X-ray crystal structure of RSK2 NTKD suggests that non-canonical location of βB-sheet in the N-lobe pushes the αC-helix, resulting in the activation of kinase activity by the disruption of the Lys–Glu interaction (26). Interestingly, the βB-sheet observed in RSK2 NTKD was found in the NTKD of MSK1 (27), but it is not detected in the NTKD of RSK1 in the X-ray crystal structure (28). The amino acid homology between NTKDs of human RSK2 and MSK1 shows only 54.2%. In contrast, amino acid identity between RSK1 and RSK2 is about 90.4%. These results suggest that the crystal structure of RSK2 NTKD might closely resemble with RSK1 rather than MSK1. Although novel activation mechanisms of the RSK2, NTKD, and CTKD have been separately provided by X-ray crystallography, the whole crystal structure of RSK2 including N- and CTKDs might need to understand more accurately about activation mechanism of RSK2 induced by tumor promoters.

**Table 1:** **Amino acid homology among human RSKs**.

	RSK1	RSK2	RSK3	MSK1	MSK2
RSK1	100.0	80.3	79.7	41.3	42.1
RSK2	80.3	100.0	83.4	42.5	42.2
RSK3	79.7	83.4	100.0	42.9	41.1
MSK1	41.3	42.5	42.9	100.0	63.5
MSK2	42.1	42.2	41.1	63.5	100.0

*The homology of amino acids was compared with each isoform of human RSKs. The sequences were obtained from human protein reference database, http://www.hprd.org*.

**Table 2:** **Amino acid homology among RSK orthologs between human and mouse**.

Mouse/human	RSK1	RSK2	RSK3	MSK1	MSK2
RSK1	96.7	80.5	79.7	42.7	40.9
RSK2	80.5	99.9	83.6	44.8	42.8
RSK3	77.8	83.6	95.6	44.3	39.6
MSK1	41.4	42.0	41.6	96.6	64.0
MSK2	41.0	42.5	42.9	66.6	95.7

*The homology of amino acids was compared with each isoform of RSK orthologs between human and mouse. The amino acid sequences of mouse RSKs were obtained from National Center for Biotechnology Information, http://www.ncbi.nlm.nih.gov/protein*.

## RSK2 Activation Mechanism and Signaling Pathway

Elucidating the RSK activation mechanism is quite challenging because of diversities of RSK nature, consecutive regulation by phosphorylation, agonist-specific temporal regulation, changing spatial distribution, existence of several interacting proteins, and having two unique kinase domains in a polypeptide. By the stimulation of cells with growth factors, cytokines and environmental stresses such as UV, RSKs are phosphorylated by multiple kinases at multiple serine and threonine residues. Notably, these sequential events are initiated by the activation of the ERK/MAPK cascade (29). For example, the epidermal growth factor receptor (EGFR) is a cytoplasmic transmembrane receptor of tyrosine kinase family involved in the regulation of the proliferation, motility, and differentiation in a variety of cell types (30, 31), and is highly expressed in cancer (32). EGF and 12-*O*-tetradecanoylphorbol-13-acetate (TPA) are well-known tumor promoters inducing malignant cell transformation in *ex vivo* cell culture and *in vivo* animal models (33). Any of these two agents can induce activation of the transcription factor activator protein-1 (AP-1) (34, 35). When JB6 Cl41 mouse skin epidermal cells are stimulated with TPA or EGF, the cells show an induction of anchorage-independent cell transformation in soft agar by AP-1’s transcriptional activation in promotion-sensitive (P+) phenotype of JB6 Cl41 cells but it is not found in promotion-resistant (P−) phenotype of JB6 Cl41 cells (36). Blocking of the TPA- or EGF-mediated AP-1 activation causes P+ cells to revert to the P− phenotype, indicates that a unique requirement for AP-1 activation in EGF- or TPA-induced cell transformation (37).

**Figure 2:**
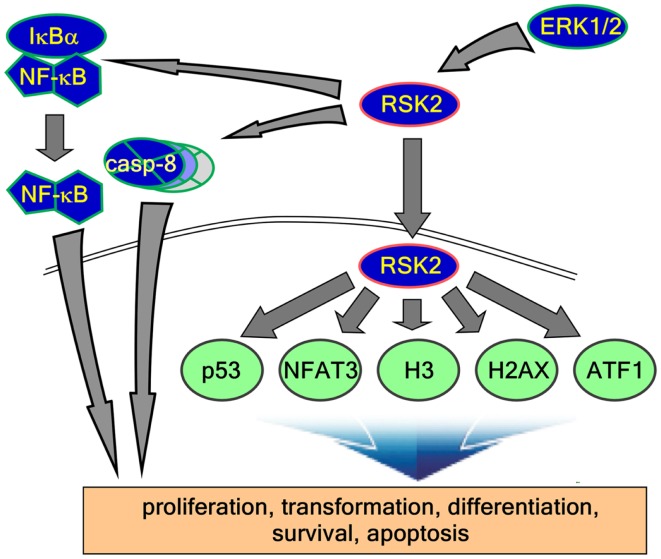
**RSK2 substrates and function of signaling axis**. Activated RSK2 by ERK1/2 transfers the phospho-signal to IκBα and caspase-8, results in the regulation of inflammation and apoptosis. On the other hand, activated RSK2 localizes into the nucleus and phosphorylates their substrates such as p53, NFAT3, histone H3, histone 2AX, ATF1, and other substrates. Taken together, these functional signaling pathways regulate diverse biological activities including cell proliferation, transformation, differentiation, survival, and cancer development.

**Figure 3:**
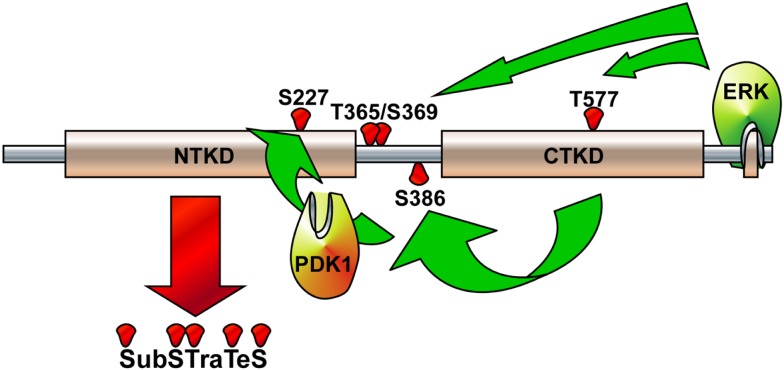
**Illustration of RSK2 activation mechanisms**. The activated extracellular signal-regulated kinase (ERK) binds at the ERK docking consensus sequences in the C-terminal domain of RSK2 and phosphorylates at Thr365/Ser369 in the linker domain and at Thr577 in the C-terminal kinase domain (CTKD), resulting in the activation of CTKD. The activated CTKD then phosphorylates at Ser386 in the linker domain, providing a docking site for PDK1. The PDK1 phosphorylates at Ser227 in the N-terminal kinase domain (NTKD), resulting in the activation of NTKD. The activated NTKD obtains ability to phosphorylate the downstream target proteins, which include kinases, transcription factors, and epigenetic factors.

Although the stimulations of cells by growth factors potentiate the Ras-ERK signaling pathway mediated by through intracellular signaling molecules such as protein kinase C, PI3-K, cAMP, and/or cytosolic calcium concentration (38) for the long-term processes such as synaptic plasticity and memory formation (39), general EGFR activation induces internal kinase domains by auto-phosphorylation and transduces their activation signals to Ras/MEKs/ERKs/RSK2 (38), resulting in the regulation of cell proliferation and differentiation (Figure 2) (38). RSK2 is a member of RSK family of serine/threonine kinases that respond to growth factors such as peptide hormones and neurotransmitters (38). Because RSK2 contains ERKs docking peptide at CTD (40, 41), RSK2 can be predominantly phosphorylated by ERKs, but not by p38 kinase (17). ERKs-mediated phosphorylation at CTKD (Thr577) and LR (Thr365 and Ser369) of RSK2 initiates the activation of the C-terminal kinase activity. The activated C-terminal kinase induces auto-phosphorylation at Ser386 in LR, which provides a docking site for 3-phosphoinositide-dependent protein kinase-1 (PDK1), resulting in induction of phosphorylation at Ser227 in NTKD by PDK1 (42). The N-terminal kinase activity of RSK2 is increased over 100-folds by phosphorylation compared with the basal level of non-phosphorylated RSK2 N-terminal kinase activity (42). Our research on the molecular mechanisms of RSK2 N-terminal kinase activity also proves that the phosphorylation abilities of RSK2 kinase to the substrates including NFAT3 (261–365) is acquired by the phosphorylation of RSK2 NTKD through CTKD-mediated N-terminal kinase activation mechanisms (Figure 3) (43). As complete activation of RSK2 NTKD to phosphorylate RSK2 substrates requires the PDK1-mediated phosphorylation of NTKD, external stimulations inducing RSK2 complete activation might activate both Ras/Raf/MEKs/ERKs and PI3-K/PKB signaling pathways, resulting in immediate induction of gene expression and protein synthesis. Taken together, stimulation of growth factors such as EGF induces docking of activated ERK1/2 at CTD of RSK2, resulting in activation of CTKD of RSK2 by ERK1/2-mediated phosphorylation at LR and CTKD. The activated CTKD of RSK2 induces auto-phosphorylation at LR of RSK2, resulting in appearance of the PDK1 docking site. After binding of PDK1 at the LR of RSK2, PDK1 phosphorylates and activates RSK2 NTKD to phosphorylate RSK2 substrates such as NFAT3, ATF1, histone H3 and H2.

## RSK2-Mediated Cell Transformation

The MAPK signaling pathway not only promotes cell proliferation but also mediates cell survival, and is up-regulated in various cancer cells (44). When cells are stimulated with a survival growth factor, brain-derived neurotrophic factor, RSK2 mediates phosphorylation of the proapoptotic protein Bcl-2 associated death promoter (BAD) (Ser112) (45), indicating that RSK2 is involved in the survival of neurons. Furthermore, RSK2 deficiency sensitizes the apoptosis induced by calcium ionophore compared with RSK2^+/+^ MEFs, indicated that RSK2 might play an important role in cell survival. On the other hand with RSK2-mediated cell survival, RSK2 plays an important role in cell proliferation and transformation. We proposed that RSK2, which is an ERK downstream serine/threonine protein kinase, might play a key role in cell transformation. Our research group confirms that RSK2 is responded with the stimulation of tumor promoters such as EGF or TPA in HaCaT human skin keratinocytes. For example, when HaCaT cells were starved for 24 h with 0.1% FBS-DMEM and then stimulated with EGF or TPA, phosphorylation and activation of ERK were increased at 5 min, maintained to 60 min, and decreased at 120 min after EGF or TPA treatment (16). The phosphorylation and total protein levels of RSKs including RSK2 was first detected at 5 min, gradually increased to 120 min, and then decreased at 240 min in a time-dependent manner with EGF or TPA treatment (16). Additionally, the RSK2 protein level was increased in a similar manner to ERK and RSKs. These results indicate that RSK2 is a protein kinase responding to tumor promoters and involving in tumor progression process. Another important signaling pathway to phosphorylate RSK2 at Tyr529 was identified by acidic FGF (aFGF) stimulation (46). The aFGF-mediated RSK2 phosphorylation at Ser386 is inhibited by the treatment of U0126, a specific MEK inhibitor, but not Wortmannin and LY294002, PI3-K inhibitors, indicating that aFGF-mediated RSK2 activation requires ERK2-induced C-terminal kinase activation. Importantly, direct phosphorylation of RSK2 at Tyr529 by FGFR3, a receptor tyrosine kinase, facilitates the binding of inactive ERK to ERK docking site in C-terminus of RSK2. This novel signaling pathway plays an important role in myeloma cell transformation (46). These results suggest that signaling pathways to activate RSK2 is not only simple through ERK, but also cooperative with receptor tyrosine kinase to induce full activation of RSK2 activity.

Another signaling pathway to enhance the skin carcinogenesis is UV-induced signaling pathway. As described earlier, UV irradiation is the most common environmental carcinogen and is highly associated with skin carcinogenesis (1). Although it is still controversial that growth factor induces mainly Ras-ERK signaling pathway rather than p38 kinase and JNKs (18), UV irradiation induces MAPK signaling pathways including ERKs, p38 kinase, and JNKs. The MAPK and PI3-K signaling pathways are specific signaling cascades in UVB-induced skin carcinogenesis (47, 48). By short time exposure of UV in cultured keratinocytes or skin, EGFR activation is increased, resulting in the activation of the ERKs signaling pathway (49, 50). UVB treatment significantly enhances ERKs (19) and p38 kinase signaling (8) and inhibition of ERKs and p38 kinase abrogated UVB-induced *c-fos* gene transcription and protein expression as well as AP-1 transactivation activity in human keratinocytes (8). Our recent research confirmed that UVB induced RSK2 phosphorylation at Thr577 in the CTKD by ERKs, resulting in the activation of phosphorylation of the NTKD at Ser227 (19). Activated RSK2 is localized into the nucleus (19). In the nucleus, RSK2 seems to induce the substrate phosphorylations including many transcription factors such as c-Fos as well as gene transcriptions by enhancing of *c-fos* gene expression (19), resulting in the increase of AP-1 complex formation and gene expression of downstream target genes. However, the molecular mechanisms of the RSK2 nuclear localization are yet to be studied clearly. One possible mechanism of the RSK2 nuclear localization is mediated by p53 protein, a well-known tumor suppressor. Interestingly, p53 protein is identified as a binding partner of the RSK2 (13). RSK2-mediated p53 phosphorylation is confirmed by the *in vitro* kinase assay and Western blot analysis using leukemia cells obtained from patients, who have normal RSK2 genetic allele and RSK2 deficient CLS patients (13). Importantly the notable point is UVB stimulation induces p53 phosphorylation at Ser15 (13) and RSK2 phosphorylation at Thr577 (19). RSK2-mediated p53 phosphorylation in cytoplasm induces nuclear p53 accumulation and increases RSK2 and histone H3 interaction (13). Interestingly p53 deficiency suppresses UV or EGF-induced histone H3 phosphorylation at Ser10, which is a RSK2-mediated phosphorylation site (16) involving cell proliferation and transformation (14).

The critical and physiological linkage between RSK2 and human skin cancer are made recently by the evidences that RSK2 is expressed in normal human skin tissues in epidermal and basal-cell layer (19). By the tissue array with human skin normal and skin cancer tissues, RSK2 protein levels were highly detected in cancer tissues compared with normal skin tissues (17, 19). Activated RSK2 by phosphorylation at Thr577 was significantly increased in skin cancer tissues compared with normal skin tissues (17, 19), indicating that RSK2 protein level and activation are closely related with human skin cancer development. More direct evidence was provided by the tissue array using the matched skin cancer tissues with the normal skin tissues, which was biopsied from adjacent skin cancer tissues in the same patient. Consequently, the evidences proves that the total and activated RSK2 phosphorylated at Thr577 is highly detected in cancer tissues compared with the matched normal skin tissues (17, 19). By extensive analysis with different skin cancer types in human skin cancer tissue array, SCC, BCC, and malignant melanoma (MM) contain higher activated-RSK2 protein levels compared with normal skin tissues (17, 19). Additionally, the RSK2 protein levels in various cancer cell lines such as H460, MCF-7, HCT-116, HCT-29, PC-2, Du-145, SoaS-2, A431, SK-MEL-5, SK-MEL-28, and RPMI 1640 are higher than compared with non-malignant human and mouse cells including HaCaT, JB6 Cl41, and NIH3T3 cells (17). The RSK2-induced cell proliferation potential has been proved A431 human skin epidermoid carcinoma cell, SK-ML-5, and SK-MEL-28 MM cells are inhibited by the treatment of kaempferol, a known RSK2 specific natural compound antagonized RSK2 NTKD activity, in the dose dependent manner (17). These results indicate that RSK2 activity is physiologically linked with skin cancer development in human.

The role of RSK2 in cell proliferation was further confirmed by the knockdown of RSK2 in human skin normal and cancer cells. RSK2 protein levels were varied according to cells context. For example, N/TERT-1, immortalized normal cells by over expression of telomerase, HaCaT, a premalignant human skin keratinocyte, SCC-13, a SCC cell, and SK-MEL-28, a MM cell, show endogenous different RSK2 protein levels likely as SK-MEL-28 > SCC-13 > HaCaT > N/TERT-1 (19). Effectively, RSK2 knockdown by short hairpin (sh)-RNA RSK2 (sh-RSK2) shows inhibitory effect in the pattern of the cell proliferation depended on the RSK2 protein levels. The N/TERT-1, which showed trace amount of RSK2 protein level, did not affect the cell proliferation by RSK2 knockdown (19). However, SK-MEL-28 MM cell contained the highest protein level of RSK2 shows the most significant inhibitory effect of the cell proliferation by the RSK2 knockdown. The inhibitory effect of the cell proliferation in these cells is oppositely affected with RSK2 protein levels as SK-MEL-28 > SCC-13 > HaCaT > N/TERT-1 (19). Moreover, RSK knockdown inhibits anchorage-independent cell transformation induced by tumor promoters such as EGF in HaCaT cells and anchorage-independent tumor growth of the SK-MEL-28 MM cell in soft agar (19). Thus, RSK2-mediated cell proliferation is a main progression effect of cell transformation in carcinogenesis.

**Figure 4:**
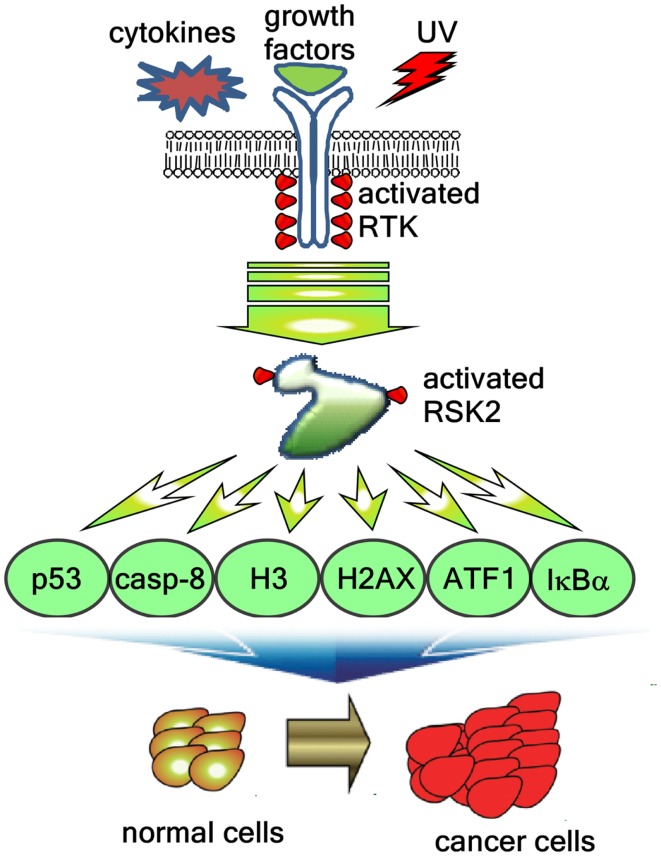
**RSK2-mediated signaling pathways regulate cancer development**. Extracellular stimuli including cytokines, growth factors, and environmental stresses induces the RSK2 phosphorylation through receptor tyrosine kinase (RTK), Ras, Raf, MEK, and ERK, resulting in activation of RSK2. The activated RSK2 phosphorylates the downstream target proteins including transcription factors such as ATF1 and p53, epigenetic factors such as histone H3 and histone 2AX, and signaling molecules such as caspase-8 and IκBα, resulting in the cell proliferation and transformation, and cancer development.

RSK2 is likely to participate in stress tolerance that can enhance the cell survival. Therefore, RSK2 is believed to stimulate cell survival (51). The understanding was made by Hemming and Klein regarding that RSKs might involve the GSK3 inhibitory phosphorylation (52, 53). GSK3 activation by dephosphorylation at serine 21 and 9 induces the cell proliferation and the cell cycle progression because the cell cycle regulating proteins including cyclin D1, C/EBP, HSF-1, NFATc, cyclic adenosine monophosphate response element-binding protein (CREB), Myc, and Jun are under the control of GSK3 (54). UVB irradiation induces the G1/G0 cell cycle phase accumulation in RSK2^−/−^ MEFs compared with RSK2^+/+^ MEFs (19). Moreover, UV-induced RSK2 phosphorylates proapoptotic BAD at serine 112, resulting in BAD activity become null and cell survival increases (55). Importantly, the RSK2 up-regulates the transcription of antiapoptotic Bcl-2 through the phosphorylation and activation of CREB (45). RSK2-mediated cell survival signaling pathway is provided by our research group that caspase-8, a critical enzyme inducing cell apoptosis, is a novel substrate of RSK2, and the phosphorylation of caspase-8 at threonine 263 by RSK2 induces caspase-8 degradation through the ubiquitination-mediated proteasomal degradation pathway (56). The net result of caspase-8 destabilization inhibits cell apoptosis, and increases cell survival and cancer development (56). These results are well consistent with our previous report that RSK2 deficiency suppresses cell proliferation through G1/G0 cell cycle arrest (16). However, mechanisms of the RSK2/GSK3 signaling pathway have not been clearly understood. Taken together, activation of RSK2 through the ERKs signaling pathway induces cell proliferation mediated by the activation transcription factors involving cell proliferation and transformation, and the suppression of the cell death signaling by BAD inactivation and caspase-8 destabilization (Figure 4).

## RSK2 Substrates and Cancer Development

The CREB is a critical regulator of immediate early gene transcription. Growth factors stimulate CREB phosphorylation at serine 133 mediated by the Ras-MAPK signaling pathway. RSK2 as a direct upstream kinase of CREB2 at serine 133 was identified *in vitro* and *in vivo* in a decade ago by Greenberg and his colleagues (57). The activation of CREB2 induces target gene expressions involved in oncogenesis including osteocalcin (58), type I collagen, TRB3, E-selectin, asparagines synthetase, and RANKL (59). ATF1 is classified as an ATF/CREB family member. Recently, our research group found that RSK2 interacted with ATF1 *in vitro* and *ex vivo*, and phosphorylated ATF1 at serine 63 (18). RSK2-mediated ATF1 phosphorylation at serine 63 enhances transactivation and transcriptional activities induced by EGF, and the point mutation of ATF1 at serine 63 to alanine 63 abolished RSK2-mediated activation effects of ATF1, resulting in inhibition of cell transformation in both Ras-induced foci formation and anchorage-independent cell transformation activities (18). Therefore, RSK2/ATF1 signaling axis plays an important role in cell transformation, especially when cells are stimulated with growth factors such as EGF.

AP-1 is a complex of Jun and Fos and it plays a key role in gene expression of target genes. The phosphorylations of c-Jun, a proto-oncogene, at serine 63 and 73 play a key role in AP-1/c-Jun transcriptional activity. Classically, the c-Jun phosphorylation at serine 63 and 73 believes through the ERKs signaling pathway because MEK inhibitors suppress the phosphorylation of c-Jun at serine 63 and 73 when cells are stimulated by growth factors such as EGF. However, after JNKs are identified as a kinase of c-Jun at serine 63 and 73, AP-1/c-Jun activation is extensively studied on the JNK signaling pathway. However, growth factor-mediated JNKs activation are still controversial because the phosphorylation of JNKs by growth factors such as EGF is minimally detected in *ex vivo* system, not likely stimulated by UV irradiation. Recently, CDK3 is identified as a c-Jun kinase at serine 63 and 73 (43). Interestingly, although murine cells widely used in *ex vivo* experiments do not contained endogenous CDK3, these murine cells still increases c-Jun phosphorylation at serine 63 and 73 by growth factor stimulation. Based on these observations, there are possibilities that some kinases might exist to phosphorylate c-Jun at serine 63 and 73 by growth factor stimulation. On the other hand, RSK2 phosphorylates C-terminus of c-Fos at serine 362, which is a critical transcription factor to regulate gene expression by formation of AP-1 complex with c-Jun, resulting in the increase of protein stability and induces osteosarcoma development (15). Therefore, RSK2 also is an important kinase to regulate AP-1 activity. However, it is not clear how c-Jun phosphorylation at serine 63 and 73 is increased by growth factor stimulation.

In carcinogenesis process, normal cells attain three important characteristics including immortalization, clonal propagation, and cellular morphology change. Especially, cellular morphology change and cell proliferation are closely related with cytoskeletal gene expression and posttranslational modification, resulting in rearrangement of cytoskeletons including lamins, keratins, neurofilaments, vimentin, desmin, and others. For example, keratin 18 can be phosphorylated at serine 52 by CAM kinase, protein kinase C and RSK2 (60). They further found that mutant serine 52 of keratin 18 induced cell cycle arrest at the G2/M stage and was correlated with histone H3 phosphorylation (60). Importantly, RSK2-mediated histone H3 serine 10 phosphorylation is confirmed by using RSK2 deficient CLS patient cells. RSK2 deficient human CLS cells are abolished the histone H3 phosphorylation at serine 10 by EGF stimulation, and re-introduction of the wild-type RSK2 to RSK2 deficient CLS patient cells restores EGF-induced histone H3 phosphorylation at serine 10 (61). Similar result is obtained by Cho and his colleagues that RSK2^−/−^ MEFs mimics histone H3 phosphorylation at serine 10 induced by EGF stimulation (16), and p53 tumor suppressor protein is involved as an intermediary protein in RSK2-mediated histone H3 phosphorylation at serine 10 when the cells are stimulated with EGF or UV (13). Although a few research groups confirmed that histone H3 phosphorylation is mainly through in MSK1, but not in RSK2, histone H3 phosphorylation at serine 10 is indispensable for the cell proliferation and transformation by stimulation of tumor promoters such as EGF (14).

Interestingly, serine 10 phosphorylation of histone H3 begins at prophase, reaches the highest level in metaphase and decreases gradually through the cell cycle to telophase (62), indicating that the histone H3 phosphorylation at serine 10 might related to chromosomal condensation process. Recently, our research group observed that histone 2B (H2B), a histone molecule containing functional redundancy with histone H3, is a new substrate of RSK2 to be phosphorylated at serine 32 (63). Although ectopic expression of the point mutant H2BS32A suppresses marginally in the cell proliferation and transformation, the phosphorylation of H2B at serine 32 was dramatically suppressed in RSK2 deficient MEFs (63). The evidence for the linkage of RSK2 and chromosome condensation is provided by which RSK2 protein is observed in G2/M phase of the developing *Xenopus* oocytes (64). These results suggest that chromatin condensation in G2/M in cell cycle phase might be regulated by RSK2 through the phosphorylation of histone 2B and histone H3. However, the molecular mechanisms in RSK2-mediated chromosome condensation have not been clearly elucidated yet.

Generally, phosphorylation of proteins is a common regulatory mechanism of protein’s activity. Nuclear factor of κ light polypeptide gene enhancer in B-cell inhibitor α (IκBα) is a key protein to regulate the activity of NF-κB, which is a key transcription factor involved in the gene expression regulation in inflammation and cancer development. The IκBα forms and inhibits NF-κB by masking the NF-κB’s nuclear localization signals (NLS), results in sequestered them in an inactive state in the cytoplasm (65). Furthermore, IKKα/β-mediated IκBα phosphorylation at serine 32 and 36 creates a binding sites for the βTrcP ubiquitin ligase complex, resulting in ubiquitination-mediated proteasomal degradation (66). By IκBα degradation, the NF-κB translocates to the nucleus and induces gene expression of many downstream target genes, including a number of antiapoptotic genes (67–69). Recently, a novel kinase against IκBα has been identified by our research group that RSK2-mediated phosphorylation of IκBα affects their protein activities by through regulation of protein stabilities (70). Similarly with IKKα/β, RSK2 phosphorylates IκBα at serine 32 *in vitro* and *ex vivo*. RSK2-mediated IκBα phosphorylation promotes the ubiquitination of IκBα, resulting in induction of p65 and p50 nuclear localization, NF-κB DNA binding, and transcriptional activity (70). More importantly, RSK2 activity inhibition with kaempferol significantly induced the apoptotic cell death when the cells were co-treated with TNF-α compared with TNF-α alone (70). These results suggest that RSK2 might play an important role in cell survival. Furthermore, caspase-8, a principal enzyme inducing apoptosis, is a novel binding partner of RSK2 and is phosphorylated at threonine 263 by RSK2 (56). Interestingly, a tumor promoter such as EGF induces caspase-8 ubiquitination and reduces half-life of caspase-8. In contrast, knockdown of RSK2 with si-RNA abrogates EGF-induced caspase-8 ubiquitination and degradation (56). These results imply that activation of RSK2 induces the activities of downstream transcription factors and inhibits apoptosis-related enzymes, which play an important role in cell proliferation and cancer development in human.

On the other hand, RSK2 substrates also include a transcription factor involved in cell differentiation, NFAT3, and apoptosis-related protein such as LKB and histone 2AX. LKB is a kinase as known as a tumor suppressor (71–73). Ectopic overexpression of LKB1 suppresses the cell growth by inducing G1 cell cycle accumulation (71). The phosphorylation of full-length LKB1 at serine 431 does not affect its kinase activity by p90^RSK^, MSK1, S6K1, and PKA. However, they found that G361 cells expressing the LKB1S431A mutant induce anchorage-independent colony growth compared with G361 cell expressing wild-type LKB1 (74). The RSK2-mediated p53 phosphorylation at serine 15 also shows noncanonical results such as inducing histone H3 phosphorylation at serine 10 and enhancing cell proliferation and transformation (13, 14). Furthermore, RSK2 also phosphorylated histone 2AX at serine 139, which is a key marker amino acid for the double-strand break (75). Astonishingly, noncanonical phosphorylation of H2AX at serine 16 and 139 by RSK2 enhances H2AX protein stability by inhibition of H2AX ubiquitination, resulting in inhibition of cell transformation. These results showed that RSK2 also regulates the cell death.

The binding of ligands to an appropriate receptor induces conformational change of estrogen receptor α (ERα), which enhances the transcriptional activation function of HBD. They further confirmed that a constitutively active mutant of RSK2 induces the phosphorylation of ERα at serine 167, resulting in enhances physical association of ERα with HBD. Importantly, anti-estrogen 4-hydroxytamoxifen blocks RSK2-mediated ERα phosphorylation by the masking of RSK2 docking site (76). These results suggest that RSK2 can regulate the cell fate depending on the cell context not only the cell proliferation and transformation, but also the cell death as summarized in Table 3. Taken together, these results suggest that RSK2 plays an important role for the determination of cell fates in both cell survival and cell death.

**Table 3:** **RSK2 substrates and cellular function in cell proliferation and transformation**.

Substrate	Phosphorylation site	Function in cancer
CREB	S133	Induction of transactivation activity
ERa	S167	Induction of transcriptional activity
Keratin 18	S53	G2/M cell cycle arrest by cytoskeleton rearrangement
Histone H3	S10	Induction of cell proliferation
NFAT3	S281/S285/S289/S334	Skeletal muscle cell differentiation
LKB1	S431	Inhibition of cell growth
IkBa	S32	Ubiquitination-mediated IkBa degradation
ATF1	S63	Induction of transactivation activity
Caspase-8	T263	Ubiquitination-mediated degradation
H2B	S32	Induction of cell proliferation and transformation
H2AX	S139/S16	Induction of protein stability and inhibition of cell transformation

## RSK2 Antagonists and Inhibitors

The ERKs signaling pathway plays a pivotal role in cell proliferation and transformation in carcinogenesis processes. Many of the human solid cancers including skin, lung, leukemia, and pancreatic cancer are genetically correlated with constitutive active Ras mutation (Ras^G12V^) (44). Thus, studies on the identification and development of novel small molecules, which can inhibit Ras-Raf-MEK-ERK signaling pathway, have rapidly expanded. Our research group has extensively studied the function of RSK2 in cell proliferation and transformation and found that RSK2 deficiency suppresses cell proliferation and anchorage-independent cell transformation compared with RSK2 wild-type MEFs (16). Further, knockdown of RSK2 with sh-RNA-RSK2 inhibits cell proliferation, EGF-induced anchorage-independent cell transformation, and colony growth of malignant human skin cancer cell in soft agar (19), indicates that RSK2 is a fascinated target to find small molecule to inhibit RSK2 activity. SL0101, Kaempferol-3-*O*-(3″,4″-di-*O*-acetyl-α-l-rhamnopyranoside), is a naturally occurring compound extracted from *Froteronica reflecta* and inhibits RSKs’ activities (77, 78). Computational docking model suggests that SL0101 binds to the ATP-binding pocket of the NTKD of RSK2 (79). In many literatures in nutrition and drug metabolism, we keenly found that glucuronized chemical compounds are cleaved in the intestinal flora before absorption and secretes to outside of body through urine and feces by drug metabolizing enzymes in the liver (80, 81). Moreover, sugar conjugated natural compounds are cleaved the sugar moiety in intestinal flora before absorption by intestinal microorganism (82). Interestingly, inhibitory effect of SL0101 in the cell proliferation about 50% was required greater amount with about 20–30 μM compared with 87 nM of IC_50_ of RSK activity *in vitro* (77–79). Therefore, our research group considered that kaempferol might an active compound in human. The hypothesis was proved that kaempferol inhibited RSK2 activity in dose dependent manner by *in vitro* kinase assay (16). Significantly, although SL0101 is showed inhibitory effects on the RSK family including RSK1, RSK2, and RSK3 (77, 78), kaempferol shows RSK2 specific inhibitory effect, but not RSK1 and RSK3, with CREB by *in vitro* kinase assay (16). The computational docking analysis of SL0101 and kaempferol suggests the differences binding modules affecting different IC_50_ level. For example, hydrogen bonds between the 5, 7-dihydroxyl moiety of SL0101 and the back bone of residues Asp148 and Leu150 in the hinge region, and extra hydrogen bonds, which occur between the ketone oxygen atom and rhamnose ring oxygen atom of SL0101. Further, they described the Thr210 hydroxyl group, between 4′-hydroxyl and rhamnose hydroxyl groups of SL0101, and the Glu197 side chain carboxylate (79), might give the stronger binding affinity between SL0101 and RSKs compared with kaempferol and RSK2 binding affinity. Our study shows to form the hydrogen bonds with Val82, Asp148, and Leu150 in the hinge region of the NTKD RSK2 (17). The absent of rhamnose ring in kaempferol might affect the IC_50_ of kaempferol about 7 μM *in vitro* compared with that of 87 nM of SL0101. However, it should give the RSK2 selectivity. Although the crystal structure of the complex of the NTKD of the RSK2 isoform with SL0101 at 1.5 Å resolution has been recently revealed (83), the crystal structure of RSK2 and kaempferol has not been reported.

BI-D1870 is a specific cell-permanent inhibitor of all RSK isoforms and it binds at the NTKD ATP-binding site. BI-D1870 inhibits the TPA-induced phosphorylation of well-known RSK substrates such as GSK3α/β and LKB1 at 10 μM concentration in response to agonists that induces the activation of RSKs (84). BI-D1870 binds at the ATP-binding pocket of the RSK2 NTKD. The compound shows the inhibition of RSK1 and RSK2 kinase activities with 10 and 20 nM concentration of IC_50_, respectively (84). However structure-based molecular inhibitory mechanisms have not been reported yet. The pyrrolopyrimidine fmk is a fluoromethylketone molecule and specifically antagonizes RSK2 activity by targeting RSK2 CTKD with 15 nM of IC_50_ and 200 nM of the half maximal effective concentration (EC_50_) (85). RSK specificity of fmk is calculated using a bioinformatics analysis of human protein kinases and found that fmk is stereoelectronically fit to the CTKD ATP-binding site. The compound induces covalent addition of chloromethylketone to thiol group of Cys436, which is located in the ATP pocket of RSK2 CTKD, resulting in the irreversible inhibition of RSK2 C-terminal kinase activities (85). The treatment of fmk inhibits partially the phosphorylation of RSK2 at Ser386 in human t (4; 14)-positive, FGFR3-expressing myeloma cell lines KMS11 and OPM (46), indicating that fmk cannot completely inhibit the RSK2 C-terminal kinase activity. Thus, fmk is a different type of RSK2 inhibitor from BI-D1870 and NTKD targeting compounds including SL0101 and kaempferol.

Staurosporine is a RSK1 specific inhibitor that binds to the ATP-binding pocket with the tetrahydropyran ring in a boat conformation. The crystal structure of staurosporine in complex with RSK1 NTKD indicates that the lactam moiety of staurosporine forms hydrogen bond to the backbone atoms of Asp142 and Leu144 of the hinge region which is similar to the RSK2-Ro31-8220 complex model. These two hydrogen bonds are complemented by an additional hydrogen bond between the *N*-methyl amino group of staurosporine and the backbone of carboxyl of Glu91 (28). The orientation of staurosporine in the ATP-binding site of RSK is similar to pose in the ATP-binding site of other important kinases such as CDK (86). By computational docking of RSK2 NTKD structure with natural compound library, our research group found that eriodictyol, a natural compound extracted from Yerba Santa (*Eriodictyon californicum*), has a similar chemical structure with kaempferol (18). Furthermore, eriodictyol docks well into the ATP-binding active site of RSK2 NTKD with −8.816 kcal/mol of binding affinity (18). Eriodictyol interacts with the backbone of Asp148 and Leu150 in the hinge region and with the Asp211 of the DFG motif in the lack cleft of the active site (18). By comparison with kaempferol docking of the RSK2 NTKD, we found that the aromatic substituent group of eriodictyol was rotated out of plane, resulting in a little weaker affinity compared with kaempferol, which was −9.216 kcal/mol of binding affinity (18). Eriodictyol suppresses RSK2-mediated ATF1 phosphorylation at serine 63, which is a novel RSK2 substrate, resulting in inhibition of cell proliferation and anchorage-independent cell transformation about 15–20 μM of IC_50_ value (18). Moreover, eriodictyol inhibits constitutive active Ras-induced foci formation (18). Thus, finding of natural compounds inhibiting RSK2 activity might be utilized as a chemopreventive agent.

## Future Direction

Previous publications demonstrated that RSK2 is an important kinase to regulate cell proliferation and transformation. Ectopic overexpression and knockdown of the RSK2 provides concrete evidences for the role of RSK2 in human skin cancer development. Moreover, our recent X-ray crystallography results of RSK2 CTKD and NTKD might support a critical analysis system to understand the molecular activation mechanisms of the RSK2 and the identification of the small molecules inhibiting RSK2 activity. Importantly, because RSK2 is a signaling molecule by protein–protein interactions and phosphate group transfer to its substrates, a remaining important issue is selectivity of inhibitors. Because many of kinases have a very similar active pocket space, which can be occupied by ATP, developing small molecules targeting ATP-binding pocket possess unwanted inhibitory effect on the other kinases, resulting in serious adverse effects. Our research group have identified many RSK2 binding partners, including transcription factors, kinases, and cell fate regulators such as cell survival and apoptosis, by mammalian two-hybrid system assay. We believe that protein–protein interaction is more specific compared with small molecule binding to proteins in physiological system. Thus, the direction to identify the RSK2 selective inhibitors might target on both the active pocket and interface between RSK2 and substrates of protein–protein interaction. With RSK2 small molecule inhibitor, the early diagnosis of the particular cancers can also be considered with total- and activated-RSK2 protein levels. For example, our previous results demonstrated that RSK2 total- and activated-protein levels were highly detected in cancer tissues compared with normal tissues. This evidence also supported with results obtained from various human cancer cell lines compared with normal or premalignant cell lines. Moreover, total- and phosphorylated levels of RSK2 was higher than that of normal tissues biopsied from adjacent tissues of cancer tissues in the same patient. Taken together, these results indicate that more intriguing studies on the RSK2 are necessary to develop as a biomarker for particular human cancers such as human skin cancer.

## Conflict of Interest Statement

The authors declare that the research was conducted in the absence of any commercial or financial relationships that could be construed as a potential conflict of interest.
